# *Tc1*-like Transposase *Thm3* of Silver Carp (*Hypophthalmichthys molitrix*) Can Mediate Gene Transposition in the Genome of Blunt Snout Bream (*Megalobrama amblycephala*)

**DOI:** 10.1534/g3.115.020933

**Published:** 2015-10-02

**Authors:** Xiu-Ming Guo, Qian-Qian Zhang, Yi-Wen Sun, Xia-Yun Jiang, Shu-Ming Zou

**Affiliations:** Key Laboratory of Freshwater Aquatic Genetic Resources, Shanghai Ocean University, Huchenghuan Road 999, Shanghai 201306, China

**Keywords:** Tc1-like transposase, transposition, Hypophthalmichthys molitrix, Megalobrama amblycephala

## Abstract

*Tc1*-like transposons consist of an inverted repeat sequence flanking a transposase gene that exhibits similarity to the mobile DNA element, *Tc1*, of the nematode, *Caenorhabditis elegans*. They are widely distributed within vertebrate genomes including teleost fish; however, few active *Tc1*-like transposases have been discovered. In this study, 17 *Tc1*-like transposon sequences were isolated from 10 freshwater fish species belonging to the families Cyprinidae, Adrianichthyidae, Cichlidae, and Salmonidae. We conducted phylogenetic analyses of these sequences using previously isolated *Tc1*-like transposases and report that 16 of these elements comprise a new subfamily of *Tc1*-like transposons. In particular, we show that one transposon, *Thm3* from silver carp (*Hypophthalmichthys molitrix*; Cyprinidae), can encode a 335-aa transposase with apparently intact domains, containing three to five copies in its genome. We then coinjected donor plasmids harboring 367 bp of the left end and 230 bp of the right end of the nonautonomous silver carp *Thm1 cis*-element along with capped *Thm3* transposase RNA into the embryos of blunt snout bream (*Megalobrama amblycephala*; one- to two-cell embryos). This experiment revealed that the average integration rate could reach 50.6% in adult fish. Within the blunt snout bream genome, the TA dinucleotide direct repeat, which is the signature of *Tc1*-like family of transposons, was created adjacent to both ends of *Thm1* at the integration sites. Our results indicate that the silver carp *Thm3* transposase can mediate gene insertion by transposition within the genome of blunt snout bream genome, and that this occurs with a TA position preference.

Transposable elements (TEs) can move within their host genomes by changing their positions of insertion in a process called transposition (McClintock 1953; Rubin and Spradling 1982; Craig *et al.* 2002). Their wide distribution among all major branches of life, their diversity, and their intrinsic biological features have made TEs a considerable source of many genetic innovations during species evolution (Radice *et al.* 1994; Kazazian 2004; Feschotte and Pritham 2007; Janicki *et al.* 2011). DNA transposons constitute one of the two subclasses of Class II TEs (Craig 1995). In eukaryotic genomes, DNA transposons have been divided into at least 17 superfamilies on the basis of sequence similarities between their element-encoded transposases (Yuan and Wessler 2011). The transposition of DNA transposons follows the “cut-and-paste” mechanism and is catalyzed by functional transposase, and allows the transposon to excise itself from a donor chromosomal site and then to reinsert at a different chromosomal locus (McClintock 1953; Kidwell and Lisch 2000).

*Tc1* transposon was first discovered in the nematode genome (*Caenorhabditis elegans*) and was shown to encode an active transposase (Emmons and Yesner 1984; Eide and Anderson 1985). *Tc1*-like transposons are approximately 1.3 kb long and contain a single gene without an intron encoding an ∼350-amino acid transposase. The transposase gene is flanked by inverted terminal repeats (ITRs) and is characterized by a DNA binding domain and a catalytic domain that can catalyze the DNA cleavage and TA dinucleotide target-joining steps of transposition (Plasterk *et al.* 1999). To date, six autonomously active *Tc1*-like transposons have been isolated from eukaryotes, *i.e.*, *Minos* from *Drosophila hydei* (Franz and Savakis 1991), *Mos1* from *Drosophila mauritiana* (Medhora *et al.* 1991), *Famar1* from *Forficula auriculata* (Barry *et al.* 2004), *Mboumar-9* from *Messor bouvieri* (Munoz-Lopez *et al.* 2008), and *Passport* from seawater flatfish (*Pleuronectes platessa*) (Clark *et al.* 2009). Moreover, four active *Tc1*-like elements have been reconstructed according to the bioinformatic consensus sequences, *i.e.*, *Sleeping Beauty* (*SB*) from salmonid (Ivics *et al.* 1997), *Frog Prince* (*FP*) from *Rana pipiens* (Miskey *et al.* 2003), *Himar1* from *Haematibia irritans* (Lampe *et al.* 1996), and *Hsmar1* from humans (Robertson and Zumpano 1997). These works have shown the ability of *Tc1*-like transposases to catalyze transposition in a variety of cells and species.

DNA transposons can be developed as valuable tools for genetic analyses (Izsvak *et al.* 2000; Kawakami *et al.* 2004; Kotani and Kawakami 2008). Identification of fish-derived *Tc1*-like transposase will provide techniques for transgenesis and insertional mutagenesis and might provide some clues to explain the fish genome evolution (Hickman *et al.* 2005). In teleost fish, *Tc1*-like transposons are found to distribute widely within the genomes. For instance, between 6% and 10% of the genome of Atlantic salmon (*Salmo salar*) is occupied by *Tc1*-like and *PiggyBac* subfamily transposons (Davidson *et al.* 2010), and approximately 4.2% of the genome of channel catfish (*Ictalurus punctatus*) is composed of *Tc1*-like transposons (Nandi *et al.* 2007). Similarly, the *Tc1*-like transposon accounts for nearly 1.7% of the genome of common carp (*Cyprinus carpio*), which is second only to that of the *hAT* transposon superfamily (approximately 2.3%) (Wang *et al.* 2011). However, the majority of *Tc1*-like transposons that have been identified in teleost fish are defective because they contain frameshifts, insertions/deletions, and premature termination codons within the coding regions of their transposase genes during their evolution (Lohe *et al.* 1995).

Because multiple copies of *Tc1*-like transposons have accumulated in the genomes of freshwater fish during evolution (Davidson *et al.* 2010; Nandi *et al.* 2007; Wang *et al.* 2011), the goal of this study is to screen a few fish *Tc1*-like elements to see whether any copy may still retain transposition function. On the basis of 17 novel *Tc1*-like transposon sequences isolated from 10 freshwater fish species, we show that one of these can encode a transposase with apparently intact domains, which can mediate DNA transposition of *cis*-element of silver carp *Thm1* in another fish species.

## Materials and Methods

### Experimental animals

A total of 18 freshwater fish species belonging to seven families were sampled for PCR analysis during different years from 2011 to 2014 (see Table 1 for details). The common carp, crucian carp, spotted steed, topmouth gudgeon, silver carp, grass carp, blunt snout bream, Fugu, yellowhead catfish, and Chinese longsnout catfish were collected at Qingpu Quanjie Fish Farm, Shanghai, China. The medaka, Nile Tilapia, Russian sturgeon, goldfish, common angelfish, and Denison barb were obtained from the Aquaculture Center of Shanghai Ocean University, Shanghai, China. The brown trout and naked carp were obtained at the Yadong Fish Farms, Tibet, China. The tail fin of each fish was cut and stored in 95% ethanol at −25°. All experiments were conducted following the guidelines approved by the Shanghai Ocean University Committee on the Use and Care of Animals.

**Table 1:** The sequence results of PCR amplifications using a single *Tc1*-like A primer

Family	Species (Sampling and PCR Analysis Year)	No. of *Tc1-like* Sequences	*Tc1-like* Elements (Designation, GenBank Acc. No.)
Cyprinidae	Common carp, *Cyprinus carpio* (2013)	961 bp, 1938 bp	1110 bp (*Tcc2*, KJ742725), 1473 bp (*Tcc3*, KJ742726), 1238 bp (*Tcc4*, KJ742727)
	Crucian carp, *Carassius auratus* (2013)	—	1473 bp (*Tca2*, KJ742724)
	Spotted steed, *Hemibarbus maculates* (2011)	1420 bp	—
	Silver carp, *Hypophthalmichthys molitrix* (2011)	1091 bp, 1020 bp	1555 bp (*Thm1*, KJ742716), 1390 bp (*Thm2*, KJ742717), 1209 bp (*Thm3*, KJ742718)
	Naked carp, *Gymnocypris przewalskii* (2011)	2036 bp	961 bp (*Tgpp1*, KJ742728)
	Grass carp, *Ctenopharynodon idellus* (2012)	—	1433 bp (*Tci1*, KJ742720), 1473 bp (*Tci2*, KJ742721)
	Blunt snout bream, *Megalobrama amblycephala* (2013)	—	1329 bp (*Tma2*, KJ742719)
	Topmouth gudgeon, *Pseudorasbora parva* (2013)	—	—
	Denison barb, *Puntius denisonii* (2013)	1116 bp, 1176 bp	1345 bp (*Tpd1*, KJ742722), 1156 bp (*Tpd2*, KJ742723)
	Goldfish, *Carassius auratus auratus* (2011)	—	—
Adrianichthyidae	Medaka, *Oryzias latipes* (2012)	1210 bp	1450 bp (*Tmf1*, KJ742729)
Cichlidae	Nile Tilapia, *Oreochromis niloticus* (2011)	1432 bp	—
	Common angelfish, *Pterophyllums carale* (2012)	1752 bp, 1465 bp, 1528 bp	1171 bp (*Tpc1*, KJ742730)
Tetraodontidae	Fugu, *Takifugu obscurus* (2012)	1044 bp, 907 bp, 1089 bp	—
Acipenseridae	Russian sturgeon, *Acipenser gueldenstaedti* (2012)	1301 bp	—
Salmonidae	Brown trout, *Salmo trutta fario* (2011, 2014)	—	1607 bp (*Tbt1*, JQ782179), 1473 bp (*Tbt2*, JQ782178)
Bagridae	Yellowhead catfish, *Pelteobagrus fulvidraco* (2012)	—	—
	Chinese longsnout catfish, *Leiocassis longirostris* (2012)	—	—

### Analysis of genomic DNA

Total genomic DNA was isolated from the tail fin samples (0.1 to 0.2 g) using a standard phenol-chloroform procedure as detailed by Sambrook *et al.* (1989). Two microliters of genomic DNA was used as the template in PCR amplification with a single primer *Tc1*-like A: 5′-TACAGTTGAAGTCGGAAGTTTACATAC-3′, which was designed according to the inverted repeats of *Tc1*-like transposon (Izsvak *et al.* 1995; Liu *et al.* 2009; Nandi *et al.* 2007). PCR reaction conditions were as follows: predenaturation at 94° for 5 min; 35 cycles of denaturation at 94° for 30 sec, annealing at 51° for 30 sec, and elongation at 72° for 2 min; and a final elongation step at 72° for 10 min. PCR products were gel-purified, ligated into the T/A cloning vector pMD-19T (Takara, Dalian, China), and transformed into *Escherichia coli DH5*α competent cells. Positive clones were examined by PCR and direct sequencing.

### Sequence analysis

The nucleotide sequences of *Tc1*-like transposons were analyzed using BioEdit 7.0.0.1 (Hall 1999). The complete inverted terminal repeat (ITR) sequence and the transposase open reading frame were filtered by comparing the left and right end ITR sequence and the transposase open reading frame region. The sequences of *Tc1*-like transposon from different fish species were obtained using the BLASTN NCBI (http://ncbi.nlm.nih.gov) search program. Alignment of the putative amino acid sequences of the *Tc1*-like transposases was performed with the Clustal X 1.81 program (Thompson *et al.* 1997). After eliminating positions with gaps, the gene genealogies were assessed by maximum likelihood using the software package PAUP*4.0b10 (Swofford 2003). For maximum likelihood, the best model and parameters were estimated by MrModeltest v2.2 (Nylander *et al.* 2004) based on the Akaike Information Criterion (AIC) and the following parameters: 500 bootstrap replications; ConLevel = 50; search = heuristic; brlens = yes.

### Southern blot analysis

The 625-bp silver carp *Thm3* probe was designed with primer pairs Thm3-f: 5′- AGATGCTGGCTGAAACTGGTAA- 3′ and Thm3-r: 5′-ATCAGGTTTGTAAGCCCTTCTC G-3′, which cover the major open reading frame (ORF) from 425 bp to 1049 bp of *Thm3*. The probes were digoxin-labeled *in vitro* using the DIG-High Prime DNA Labeling and Detection Starter Kit I (Roche, Basel, Switzerland) according to the manufacturer’s instructions. Southern blot analysis was performed as described previously by Koga *et al.* (2000), with the following modifications. High-molecular-weight DNA (20–30 µg for each gel slot) were digested with *Bgl*II restriction enzyme, fractionated on 0.8% agarose gels, and transferred to nylon membranes (Hybond-N; Amersham), followed by fixation via baking at 80°. The membranes were then hybridized with a digoxin-labeled silver carp *Thm3* probe. Finally, stripping and reprobing DNA blots were performed after immunological detection.

### Plasmid constructs

To generate the pCS2-CMV-Thm3TP construct, the ORF of the silver carp *Thm3* transposase cDNA, which encodes 335 aa residues, was amplified by PCR using *Pfu* DNA polymerase (Stratagene, La Jolla, CA, USA) and the primers 5′-CCGCTCGAGATGGGAA AATCAAGAGAA-3′ and 5′-GCTCTAGATTAGTATTTGGTAGAATT-3′. This was then digested with *Xho*I and *Xba*I and subcloned into the *Xho*I*-Xba*I sites of the pCS2+ vector. The sequences of all plasmids were confirmed by DNA sequencing. The left (367 bp) and right (230 bp) *Thm1* inverted terminal repeats (ITRs) were amplified from the silver carp using PCR primers for the left ITRs (sense, 5′-GGACTAGTTACAGTTGAAGTCGGAAG-3′; antisense, 5′-CCGCTCGAGCTCTGTGTTTGAGGTGTG-3′) and right ITRs (sense, 5′-GAAGATCTGAAGTGTATGTATACTTCTGACTT-3′; antisense, 5′-GGGGTACCTACAGTTGAAGTCGGAAGTTTACA-3′). To generate the pThm1-Mlyz2-RFP (red fluorescent protein) construct, the left ITR PCR product was digested with *Spe*I and *Xho*I, and the right ITR PCR product was digested with *Bgl*II and *Kpn*I. These two fragments were then subcloned into the *Spe*I*-Xho*I and *Bgl*II*-Kpn*I sites, respectively, of pTgf2-Mlyz2-RFP that contained the zebrafish myosin light chain 2 (*Mlyz2*) gene promoter (Guo *et al.* 2013).

### *In vitro* transcription and microinjection

Capped silver carp *Thm3* transposase mRNA were synthesized *in vitro* using the mMESSAGE mMACHINE kit (Ambion, Austin, TX) using *Not*I linearized pCS2-CMV-Thm3TP plasmid DNA as a template. The microinjection volume was estimated at 1 nL/embryo and contained 50 pg of circular donor plasmid pThm1-Mlyz2-RFP coinjected with 50 pg of *Thm3* transposase capped mRNA. This was injected into blunt snout bream embryos at the one- to two-cell stage. The linearized donor plasmid pThm1-Mlyz2-RFP was injected without *Thm3* transposase capped mRNA as a control. After injection, the embryos were placed in an embryo rearing medium and maintained at room temperature. Fluorescent expression in the embryos was analyzed using a Nikon SMZ1500 fluorescence microscope.

### Transgene efficiency and insertion site analysis

The primers used for RFP PCR were: RFP-f, 5′-GCATGGAGGGCTCCGTGAACG-3′ and RFP-r, 5′-GGTGTAGTCCTCGTTGTGGG-3′. PCR amplification, cloning, and sequencing were conducted as previously described (Jiang *et al.* 2012). The flanking sequences of the transposon insertion sites were analyzed using the GenomeWalker Universal Kit (Clontech, California). For splinkerette PCR, 25 µg genomic DNA was digested for 12–16 hr at 37° with 80 units of *Stu* I and *EcoR* V in a 100-µL reaction volume, purified by ethanol precipitation, and 4 µL of the digestion mix was ligated with the splinkerette adaptor overnight at 16°. The linker ligation was used as a template for two rounds of PCR to amplify the transposon/genome junction. The nested primers for the 5′ flanking sequences were 5′-AGGTGTGCCTTTATATTCATCCACAGGCGT-3′ and 5′-ATCAATTAACCTATCAGAAGCTTCTT-3′. The nested primers for the 3′ flanking sequences were 5′-TAAGGAAGTGTATGTATACTTCTGACTTTGA-3′ and 5′-TCCCTCGCTCGATTCTGACATTTAACA-3′. The amplified fragments were cloned into the pMD19-T vector (TaKaRa, Dalian, China) and transformed into *DH5α E. coli* cells, and the positive clones were examined by PCR and direct sequencing.

### Data availability

Figure S1 contains supporting figures. Further information on all plasmids and more detailed supporting data are available upon request.

## Results

### PCR screening and sequence analysis of Tc1-like elements from different fish species

A total of 34 specific nucleotide sequences (ranging from 907 bp to 2036 bp) were amplified from the 18 fish species using the single *Tc1*-like A primer complementary to ITRs (Table 1). Seventeen of these sequences exhibited structural features of the *Tc1*-like transposon comprising the ITRs and the transposase coding region. The remaining 17 sequences coded for other genes or were unknown sequences. The 17 *Tc1*-like transposons were distributed in 10 species belonging to four families (Table 1). Like most members of the *Tc1*-like family, the 17 cloned *Tc1*-like transposons were always flanked by a TA target site (data not shown). All *Tc1*-like transposon sequences we obtained are available in GenBank under Accession numbers KJ742716–KJ742730. Because brown trout *Tbt1* and *Tbt2* cloned from the samples collected in 2014 had >99% similarities to the sequences identified in 2011 (Guo *et al.* 2014), previous GenBank accession numbers (JQ782178–JQ782179) were used for both sequences in this study (Table 1).

**Figure 1:**
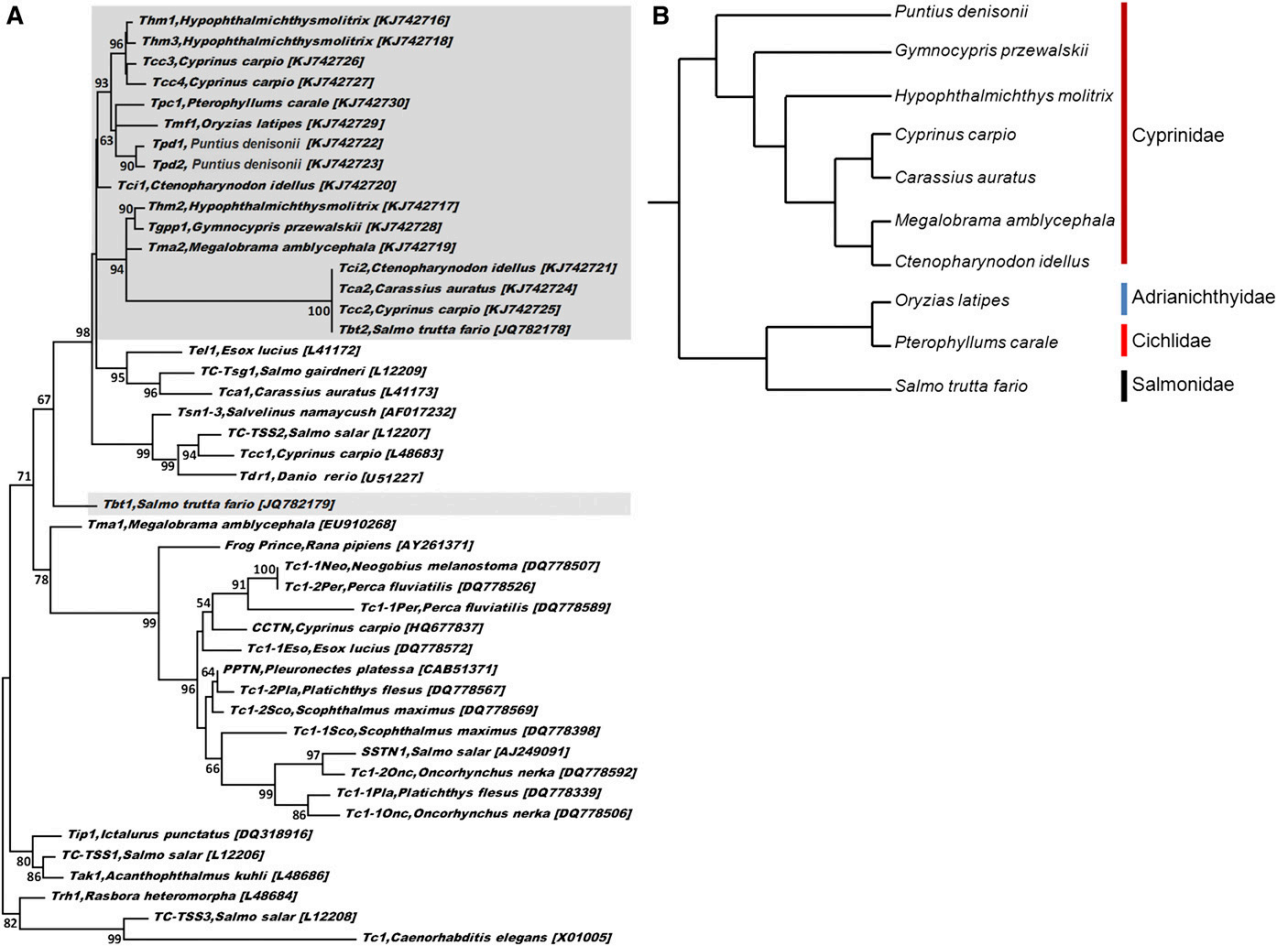
(A) Maximum likelihood (ML) consensus tree for *Tc1*-like transposases from a variety of teleost fish species. Bootstrap percentages are shown by numbers at the interior nodes. The *C. elegans Tc1* sequence was used as the outgroup. The isolated 17 *Tc1*-like transposon sequences of this study are shown in gray in the background. (B) Phylogenetic hypotheses proposed by Nelson (2006) and Wang *et al.* (2007) for fish species in this study.

We used the 17 cloned *Tc1*-like transposons DNA sequence as queries for BLAST n searches of the GenBank databases; all sequences matched similarity above 50% were used for phylogenetic analysis. Our results indicate the *C. elegans Tc1* does not appear as a monophyletic group, but clusters together with *TC-TSS3 Salmo salar* as outgroup with a high bootstrap value (Figure 1A). The isolated *Tc1-like* sequences seem to cluster as a distinct subfamily of the *Tc1*-like transposon with 98% of bootstrap, except for brown trout *Tbt1*. As shown in Figure 1A, the *Tpd1* and *Tpd2* from Denison barb do not cluster to the *Tc1*-like sequences from other Cyprinidae fish species, but cluster well with the medaka *Tmf1* from Adrianichthyidae and the common angelfish *Tpc1* from Cichlidae. Even for the isolated 13 *Tc1*-like sequences from seven species of the Cyprinidae family, their phylogenetic relationship is patchy clustering (Figure 1A). This means that these Cyprinidae *Tc1*-like sequences are not in line with the vertical evolutionary relationships (Figure 1B). By pairwise comparisons, *Tc1*-like transposons of the same size (1473 bp) isolated from grass carp (*Tci2*), common carp (*Tcc2*), crucian carp (*Tca2*), and brown trout (*Tbt2*) shared >99% identity to quite distantly related groups (Figure 1A; Table 1). The closely related *Tc1*-like transposons described here are distributed among divergent lineages of Cyprinidae and Salmonidae (diverged for >250 million years). The patchy distribution *Tc1*-like elements coupled with the extreme level of similarity between *Tc1*-like elements in the distantly divergent host species are incompatible with vertical inheritance, but are strongly indicative of multiple horizontal introductions.

**Figure 2:**
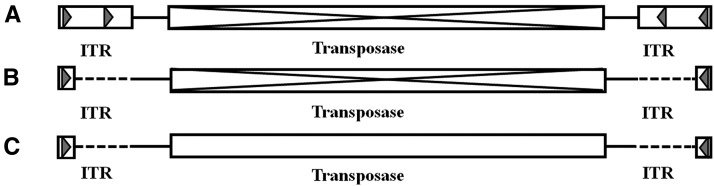
Analysis of inverted terminal repeat sequences (ITRs) and coding regions of *Tc1*-like transposon from different fish species. Patterns contain (A) complete left and right ITRs and an interrupted transposase, (B) incomplete left and right ITRs and an interrupted transposase, and (C) incomplete left and right ITRs and an intact transposase.

The 17 *Tc1*-like elements screened from different fish species could be divided into three structural patterns based on their ITRs and their transposase ORF. One pattern involved silver carp *Thm1*, naked carp *Tgpp1*, and brown trout *Tbt1*, and preserved complete left and right ITRs, whereas the transposase regions were interrupted by frameshifts or stop codons (Figure 2A). A second pattern involved silver carp *Thm2*, grass carp *Tci1*, *Tci2*, Denison barb *Tpd1*, *Tpd2*, crucian carp *Tca2*, common carp *Tcc2*, *Tcc4*, medaka *Tmf1*, common angelfish *Tpc1*, and brown trout *Tbt2*, which had incomplete ITRs as well as interrupted transposases (Figure 2B). The third pattern involved silver carp *Thm3*, common carp *Tcc3*, and blunt snout bream *Tma2*, which have lost some nucleotides in the ITRs, but have preserved uninterrupted transposases encoding 335 aa, 280 aa, and 238 aa *Tc1*-like transposases, respectively (Figure 2C).

### Uninterrupted Tc1-like transposase Thm3 screening from silver carp

**Figure 3:**
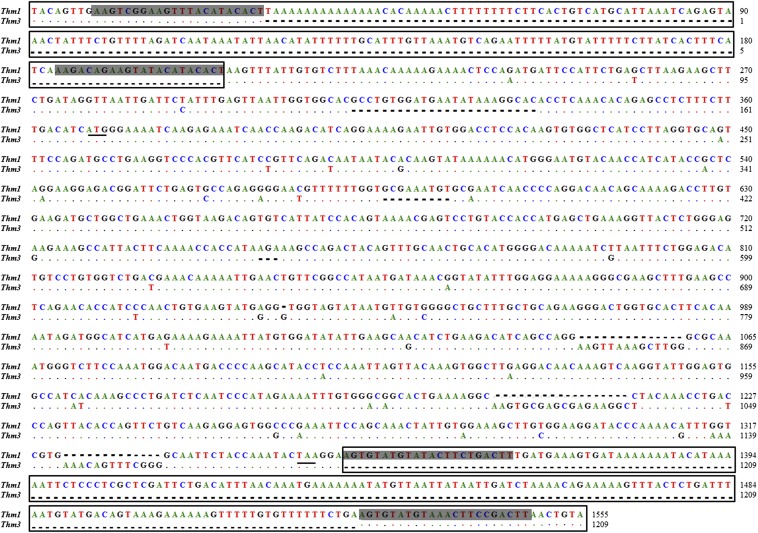
Sequence alignment of *Thm3* with *Thm1* from silver carp. The ITRs are shown in open boxes. The gray background sequences represent internal repeats/directed repeats (IRs/DRs). Dashed lines represent missing nucleotides.

**Figure 4:**
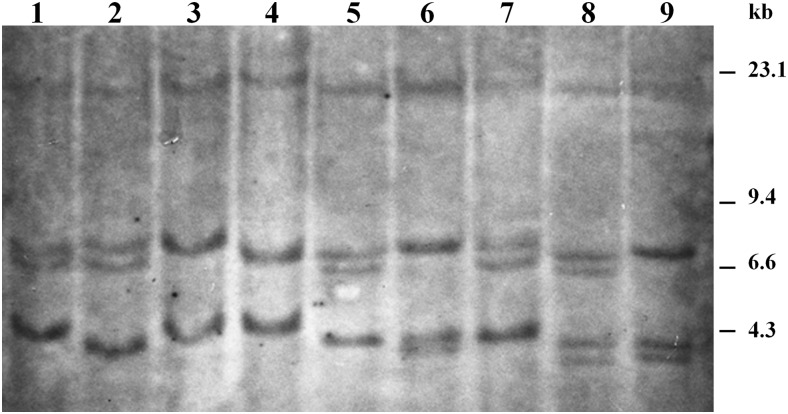
Southern blot hybridization analysis of the *Thm3* transposon in the silver carp genome. Genomic DNA of nine individuals was sampled from the tail fins of silver carp. Genomic DNA was digested with *Bgl*II and hybridized with a digoxin-labeled *Thm3* probe. The marker sizes are indicated along the right margin.

The ORF of silver carp *Thm3* encodes 335 amino acids of the transposase but preserves incomplete ITRs (Figure 3). Meanwhile, the silver carp *Thm1* has long ITRs of ∼210 bp with internal repeats/directed repeats (IRs/DRs), an internal structure described by Izsvak *et al.* (1995), but preserves interrupted transposases. The silver carp *Thm3* and *Thm1* elements share a high similarity (91%), except for some insertion/deletion nucleotides (Figure 3). Our further Southern blot analyses reveal that there are only three to five copies of the *Thm3* transposon in the silver carp genome (Figure 4). However, the positions of these *Thm3* copies are relatively constant in the silver carp genome, which indicates the silver carp *Thm3* transposase may lose its autonomous transposition activity. Although this may also be attributed to the endogenous inhibition of host cell (Castaneda *et al.* 2011), our efforts to detect mRNAs encoded by the *Thm3* element indeed failed to demonstrate the presence of the putative transposase in multiple tissues of silver carp (data not shown).

**Figure 5:**
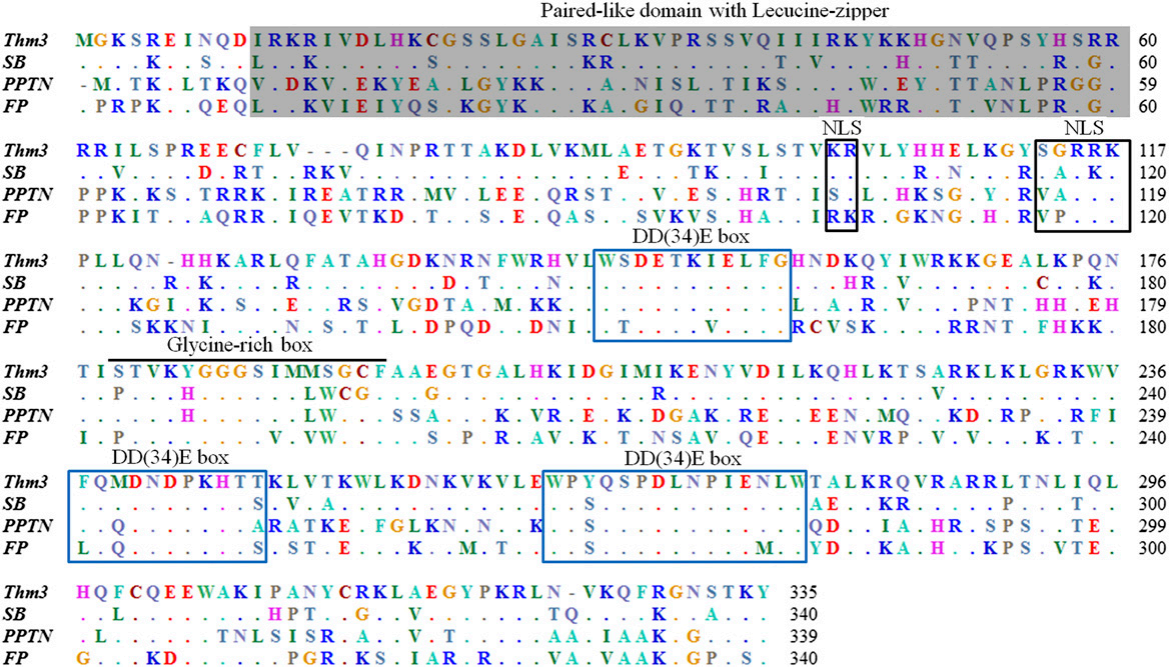
Alignment of the amino acid sequences of *Tc1*-like transposases from the silver carp *Thm3*, reconstructed transposase *SB* from salmonid, reconstructed transposase *FP* from *Rana pipiens*, and natively active *PPTN* transposase from flatfish. The major functional domains are highlighted according to the structural domains of the *SB* transposase (Ivics *et al.* 1997).

The 335-aa *Thm3* transposase from silver carp exhibits greater than 80% sequence identity with reconstructed transposase *SB* from salmonids, but only 46% and 44% sequence identity with reconstructed transposase FP from *Rana pipiens* and natively active flatfish *PPTN*, respectively (Figure 5). As seen in salmonid *SB* transposase, the silver carp *Thm3* transposase contains the main functional domains of *Tc1*-like transposases, *i.e.*, a paired-like domain with leucine-zipper for DNA binding, a bipartite nuclear localization signal (NLS), a glycine-rich box, and complete DD(34)E domains in the C-terminal (Figure 5). The DD (34) E domain is the essential site for catalytic transposition of *Tc1*-like transposase. There are two Aspartates (D, D) separated by 90 amino acids, the second of which is separated from Glutamate (E) by 34 amino acid residues (Figure 5). The silver carp *Thm3* transposase retains its intact functional domains, which suggests that it could be used to construct gene transfer systems in other teleost fish.

### Silver carp Thm3 can mediate gene transposition in blunt snout bream genome

**Figure 6:**
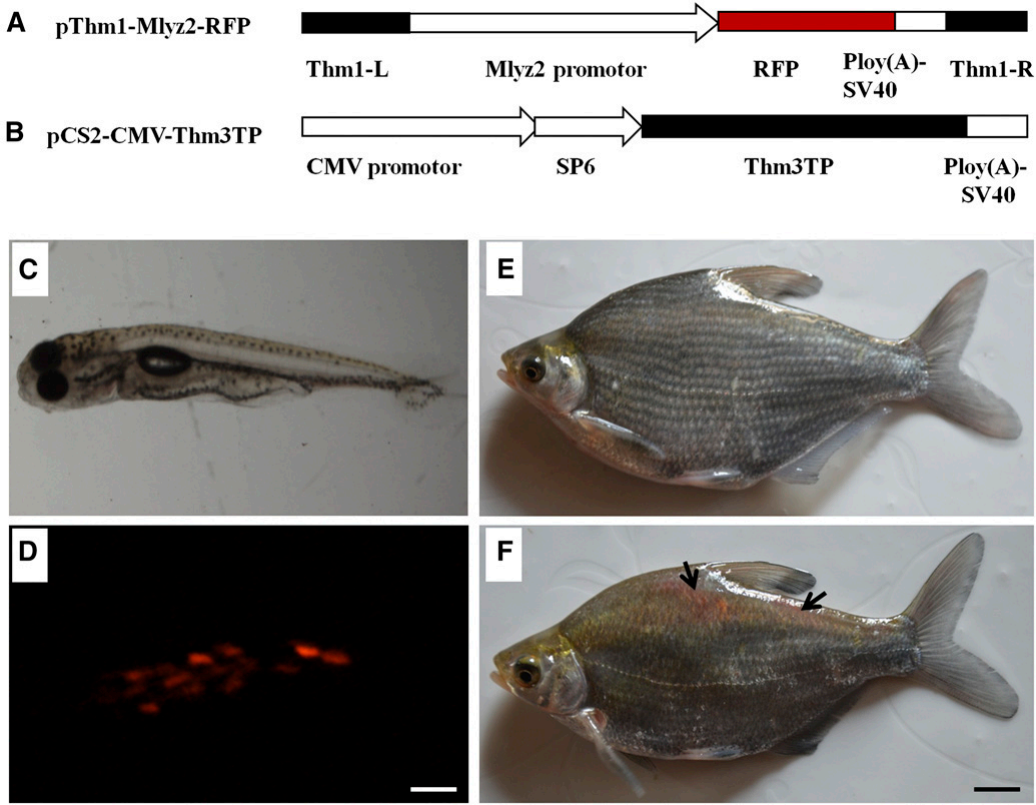
Results of transposition efficiency analysis of silver carp *Thm3* transposase in blunt snout bream. (A) The donor plasmid construct pThm1-Mlyz2-RFP harbors the left (367 bp) and right (230 bp) silver carp *Thm1* inverted terminal repeats (ITRs). This also contains the zebrafish myosin light chain 2 (Mlyz2) promoter, the red fluorescent protein (RFP), and the SV40 poly(A) signal. (B) The transposase plasmid construct pCS2-CMV-Thm3TP. Capped silver carp *Thm3* transposase mRNAs were synthesized *in vitro*, using linearized pCS2-CMV-Thm3TP plasmid DNA as a template. Light (C) and fluorescence (D) microscopy images of RFP expression in blunt snout bream embryos at 72 hpf after coinjection of the donor plasmid pThm1-Mlyz2-RFP and capped silver carp *Thm3* transposase mRNAs in embryos at the one- to two-cell stage. Optical images of RFP expression in the negative control (E) and in transgenic positive individuals (F) in 200-d-old blunt snout bream. Arrows indicate RFP expression signals. White scale bars = 600 µm. Dark scale bars = 2 cm.

To explore whether the silver carp *Thm3* transposase has transposition activity, a binary transposon vector system was developed based on *Thm3* transposase and ITRs from the silver carp nonautonomous element *Thm1*. We constructed the donor plasmid pThm1-Mlyz2-RFP with a 367-bp left-end and 230-bp right-end of the *Thm1* transposon (Figure 6A). In addition, the pCS2-CMV-Thm3TP plasmid was constructed and used to *in vitro* synthesize capped RNA encoding 335 aa residues *Thm3* transposase (Figure 6B). We then coinjected 50 pg of donor plasmid DNA with 50 pg of capped RNA into the embryos (one- to two-cell stage) of blunt snout bream. We found that the red fluorescence expression was mainly focused in the myofibrils in blunt snout bream embryos at 72 hr postfertilization (hpf), indicative of tissue-specific expression by the zebrafish myosin light chain 2 (Mlyz2) promoter (Figure 6, C and D). In 200-d-old adult blunt snout bream, the red color could be observed in the dorsal skeletal muscles of transgenic individuals (Figure 6, E and F). PCR analysis of the transposition rate of RFP at adult blunt snout bream was performed as previously described (Jiang *et al.* 2012). The integration rate of RFP gene into the blunt snout bream genome when fish were 200 d old ranged from 47.0 to 59.5% (average = 50.6%, 519/1015) (Table 2). In control embryos by injected linearized donor plasmid alone, only 20.2% of embryos showed weak and high rates of the mosaic expression of RFP and few fish expressed RFP at the 200-d-old stage (data not shown). Moreover, PCR analysis showed that the average RFP-transgenic rate in control adult fish injected with the linearized donor plasmid pThm1-Mlyz2-RFP alone was 5.0% (8/184) (Table 2).

**Table 2:** The transposition efficiencies of silver carp *Thm* transposon systems in 200-d-old blunt snout bream by PCR analysis

Batches	No. of Survival Injection Individuals	No. of Individuals Integrated RFP	Integration Rates (%)
1	66	31	47.0
2	147	64	43.5
3	121	72	59.5
4	87	41	47.1
5	212	107	50.5
6	187	96	51.3
7	195	108	55.4
Average	145	74	50.6*^a^*
Control 1	115	5	4.3
Control 2	69	3	5.7
Average	92	4	5.0

Control is injected pThm1-Mlyz2-RFP plasmid only.

a *P* < 0.01.

To precisely characterize insertions at a sequence level, we cloned the junctions of the integrated *Thm1 cis*-element and the surrounding genomic DNA using inverse PCR. Because the endogenous *Tc1*-like element *Tma2* was also present in the genome of blunt snout bream (Figure 1, Table 1), its similarity with sequences of the left-end (367 bp) and right-end (230 bp) of silver carp *Thm1* were 54% and 65%, respectively (supporting information, Figure S1). To avoid false positives, we chose low-similarity (<50%) regions to design the nested primers to clone the 5′ or 3′ flanking sequences (Figure S1). Among five RFP-transgenic positive individuals, there were one to four genomic integration sites in the genome of the blunt snout bream, including the specific end sequences of *Thm1* DNA (Table 3; Figure S1), whereas no PCR products during splinkerette PCR were amplified in the genome of the negative control (data not shown). Moreover, a TA dinucleotide direct repeat of the target DNA at the integration site (which is the signature of *Tc1*-like family of transposons) was created adjacent to both ends of *Thm1* at the integration sites in all individuals (Table 3). These results indicate that the silver carp *Thm1* transposon insertions occurred by transposition mediated by the *Thm3* transposase. It also suggests that during DNA transposition the silver carp *Thm* transposon system exhibits a TA insertion position preference. Furthermore, the RFP-transgenic fish were raised to maturity and crossed with the opposite sex blunt snout bream of wild-type, and F1 embryos were analyzed for RFP expression under a fluorescent microscope at 48 hpf. Among 11 RFP-transgenic positive fish that we randomly selected, 10 of 11 founders were able to express RFP in their F1 embryos from 13 to 64% (Table 4), indicating that the silver carp transposon vector inserted into the blunt snout bream genome could be transmitted to the F1 progeny.

**Table 3:** Exogenous *Thm1* end regions and the surrounding transposon insertion site sequences in the genome of 200-d-old blunt snout bream

Sample No.	Copies	Sequence of Target Integration Sites
1	i	(143nt)GTACTTTATACAGTT——AACTGTATACAGCAT(11nt)
	ii	(180nt)CAGACTTCTACAGTT——AACTGTACTGTCACT(92nt)
	iii	(75nt)ACATATATTACAGTT——AACTGTAAATGTGTC(138nt)
	iv	(85nt)CCTTGTTATACAGTT——AACTGTATTAAATGG(175nt)
2	i	(92nt)TTATGACCTACAGTT——AACTGTATTCATATA(127nt)
	ii	(38nt)TAAGGTTGTACAGTT——AACTGTACGCCAAAC(95nt)
3	i	(28nt)AACTGCTCTACAGTT——AACTGTAACTGAGTA(134nt)
	ii	(73nt)GTTGTAGTTACAGTT——AACTGTACAGTACTA(117nt)
	iii	(92nt)GCGTAAGTTACAGTT——AACTGTATAGTAGCG(83nt)
4	i	(177nt)GGATAGTTTACAGTT——AACTGTATGAGTGAA(76nt)
5	i	(58nt)AGACTTGGTACAGTT——AACTGTAGACGATTT(154nt)
	ii	(14nt)GTAGGGTTTACAGTT——AACTGTACGGTAAAT(217nt)
	iii	(73nt)GGTATAGGTACAGTT——AACTGTAGGTATACG(312nt)

The TA dinucleotide direct repeats of the target DNA are marked in gray, and the end sequences of *Thm1* DNA are underlined.

**Table 4:** RFP expression in F1 embryos of 11 RFP-transgenic positive blunt snout bream crossed with the opposite sex of wild-type

ID of RFP-Transgenic Positive Fish*^a^*	Gender	No. of F1 Embryos Examined	No. of RFP Positive Embryos	RFP Positive/Total F1 (%)
690000116601632	Female	89	57	64
690020042302853	Female	254	123	48
690000116602112	Male	275	40	15
690020042302431	Male	212	27	13
690020042302497	Female	238	38	16
690020042303065	Male	152	78	51
690020042303056	Male	96	55	57
690020042302805	Female	321	0	0
690020042303004	Female	186	27	15
690000116601753	Female	141	51	36
690000116602026	Male	311	50	16
Control*^b^*	—	215	0	0

a The IDs of RFP-transgenic positive fish were labeled with passive integrated transponder (PIT) tags (Hongteng Barcode Corporation, Guangzhou).

b The wild-type female mating with male was used as control.

## Discussion

In this study, we isolated 17 *Tc1*-like transposons from 10 freshwater fish species, and most (16) of which clustered as a new subfamily of *Tc1*-like transposons. Like most members of the *Tc1*-like family (Eide and Anderson 1985; Emmons and Yesner 1984; Izsvak *et al.* 1995), the 17 cloned *Tc1*-like transposons were always flanked by a TA target site. Of these transposons, we have shown that the *Thm3* transposon of silver carp (*Hypophthalmichthys molitrix*) contains the main functional domains of *Tc1*-like transposases and is present at relatively low copy numbers. A binary transgene vector system was developed based on the 335-amino acid *Thm3* transposase and terminal inverted repeats (ITRs) from silver carp nonautonomous element *Thm1*. Although *Thm3* is not an autonomous transposon in the silver carp, our results show that the *Thm3* transposase can efficiently mediate gene insertion in the genome of one of its Cyprinidae relatives, the blunt snout bream (*Megalobrama amblycephala*), and can mediate transposition in the blunt snout bream germ lineage. We have shown that the silver carp *Thm* transposon system has a TA insertion position preference during DNA transposition in blunt snout bream.

DNA transposons transferred by the horizontal approach have been found in multiple prokaryotic and eukaryotic species (Leaver 2001; de Boer *et al.* 2007; Pocwierz-Kotus *et al.* 2007; Pace *et al.* 2008; Kuraku *et al.* 2012; Jiang *et al.* 2012). In the present study, the isolated *Tc1*-like sequences from different fish species do not conform to the vertical evolutionary relationships, and their phylogenetic relationship is patchy clustering. This means that the isolated *Tc1*-like transposons might be horizontally transmitted among species because of inconsistency with the accepted phylogenetic relationships of their host organisms. Also, the idea that horizontal transmission has played a role in the dissemination of cloned *Tc1*-like transposons in this study is supported by the presence of a complete copy in the fish genome. An almost identical 1473-bp *Tc1*-like transposon is found to distribute in the diverged Cyprinidae and Salmonidae, and their nucleotide sequence similarity is markedly greater than some of the most conserved protein-coding genes in vertebrates (*e.g.*, *RAG-1*) (Venkatesh *et al.* 2001). Another convincing example is seen in the transposons *Tc1-1Neo* from round goby and *Tc1-2Per* from European perch, which have virtually identical nucleotide sequences and have been found in fish belonging to quite distantly related groups (Figure 1A and see Pocwierz-Kotus *et al.* 2007). Despite the lack of direct evidence, our results suggest that horizontal transmission of *Tc1*-like transposons seems to occur in some divergent fish lineages. Interestingly, evidence has been found that horizontal transfers of *Tc1*-like elements can occur between teleost fishes and lampreys and their vertebrate parasites (Gilbert *et al.* 2010; Kuraku *et al.* 2012).

Transposons are believed to have evolved via three processes: horizontal transmission, vertical inactivation, and stochastic loss (Lohe *et al.* 1995). An autonomous DNA transposon usually encodes an endogenous functional transposase in the host cell, which is expected to have originated relatively recently via horizontal transfer (Eide and Anderson 1985; Koga and Hori 2000; Clark *et al.* 2009; Jiang *et al.* 2012). However, most transposons, especially those in teleost fish, undergo vertical inactivation or stochastic loss to produce a large number of different copies of unautonomous transposons that have mutations in their transposase regions (Leaver 2001; Ahn *et al.* 2008; Wang *et al.* 2011; Pujolar *et al.* 2013). For example, in the channel catfish genome the *Tip1* transposon exists at approximately 150 copies per haploid genome, whereas the *Tip2* transposon exists at approximately 4000 copies per haploid genome (Nandi *et al.* 2007), with 32,000 copies of the nonautonomous element *Tipnon* present (Liu *et al.* 1999). However, in the silver carp genome, our results showed that low numbers of three to five copies of *Thm3* transposon existed. This suggests that horizontal transmission of *Thm3* transposon into the silver carp genome might not be too long, and yet vertical inactivation may not occur in the host cell. In fact, the silver carp *Thm3* transposase retains its intact functional domains and it may be used to construct gene transfer systems.

The *Tc1*-like transposons are powerful molecular tools for transgenesis in vertebrate cells (Gallardo-Galvez *et al.* 2011; Garrels *et al.* 2014). Most *Tc1*-like transposons identified in fish are considered defective because they contain insertions/deletions that result in either sequence frameshifts or point mutations, which lead to a premature stop codon or a nonsense codon in the transcribed mRNA (Radice *et al.* 1994). Recently, several novel *Tc1-like* transposable elements have been found to include all the functional domains of *Tc1*-like transposons, *e.g.*, *CCTN* in the genome of the common carp, which contains an intact 331 aa *Tc1-like* transposase (Wang *et al.* 2011), *e.g.*, the *MMTS* transposon that contains motifs including DDE from mud loach (Ahn *et al.* 2008), and, *e.g.*, the *Tana1* transposase composed of 341 amino acids in the genome of sturgeons (Pujolar *et al.* 2013). However, further assays should be conducted to test if the catalytic activity of the DDE motif remains active. In this study, a binary silver carp *Thm* vector system was developed to enhance production of transgenic blunt snout bream as an alternative to methods of transgenesis involving the injection of donor plasmid DNA. We have shown that the silver carp *Thm3* transposase can efficiently catalyze the integration of donor DNA into a TA dinucleotide site of a recipient genome. In the future, it would be good to compare the efficiency of transposition of the new element with *sleeping beauty* or some previously isolated *Tc1*-like element, although these *Tc1*-like elements such as *SB* and *FP* were seldom used for fish transgenesis.

## Supplementary Material

Supporting Information
