# Supplementary material for: Tc1-like Transposase Thm3 of Silver Carp (Hypophthalmichthys molitrix) Can Mediate Gene Transposition in the Genome of Blunt Snout Bream (Megalobrama amblycephala)
Source: G3 (Bethesda). 2015 Oct 2;5(12):2601–10. doi: 10.1534/g3.115.020933 (PMC4683633; doi:10.1534/g3.115.020933)
Supplement: Supporting Information [file supp_5_12_2601__index.html]

Tc1-like Transposase Thm3 of Silver Carp (Hypophthalmichthys molitrix) Can Mediate Gene Transposition in the Genome of Blunt Snout Bream (Megalobrama amblycephala) — Supporting Information 

# *Tc1*-like Transposase *Thm3* of Silver Carp (*Hypophthalmichthys molitrix*) Can Mediate Gene Transposition in the Genome of Blunt Snout Bream (*Megalobrama amblycephala*)

## Supporting Information for Guo *et al.*, 2015

**Files in this Data Supplement:**

- Figure S1 - Sequence alignment of the 367 bp-left-end (A) and 230 bp-right-end (B) silver carp *Thm1* with blunt snout bream *Tma2*. (PDF, 675 KB)
