# Supplementary material for: Tc1-like Transposase Thm3 of Silver Carp (Hypophthalmichthys molitrix) Can Mediate Gene Transposition in the Genome of Blunt Snout Bream (Megalobrama amblycephala)
Source: G3 (Bethesda). 2015 Oct 2;5(12):2601–10. doi: 10.1534/g3.115.020933 (PMC4683633; doi:10.1534/g3.115.020933)
Supplement: Supporting Information [file supp_g3.115.020933_FigureS1.pdf]

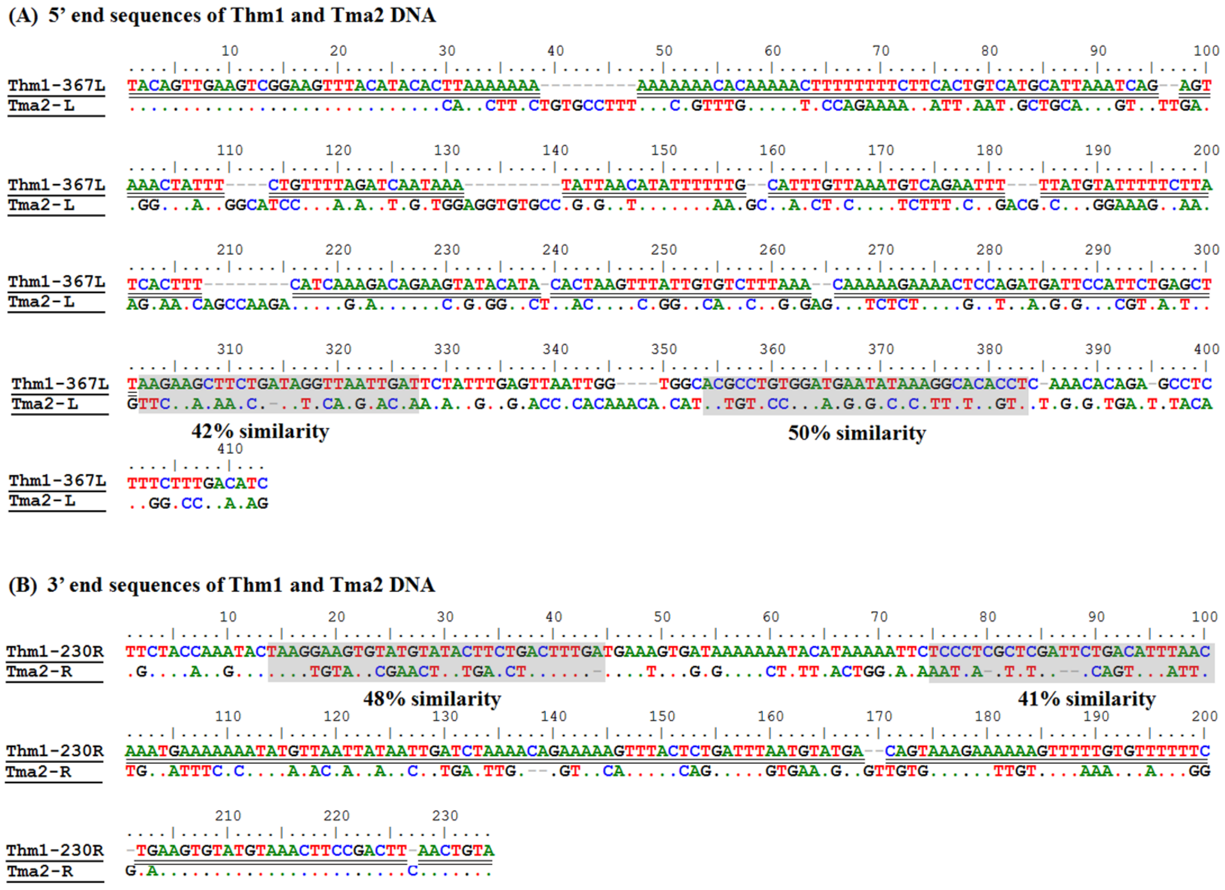

Figure S1: Sequence alignment of the 367 bp-left-end (A) and 230 bp-right-end (B) silver carp *Thm1* with blunt snout bream *Tma2*. The nested primers for the 5' or 3' flanking sequences are marked in gray. Dashed lines represent missing nucleotides and dots mean similar nucleotides. The double underlined sequences are the end sequences of *Thm1* DNA at target integration sites in the genome of the blunt snout bream as shown in Table 3.
